# Editorial: Exploring epigenetic mechanisms in cancer

**DOI:** 10.3389/fonc.2026.1718426

**Published:** 2026-01-27

**Authors:** Zaki A Sherif, Habtom W. Ressom, Olorunseun O Ogunwobi

**Affiliations:** 1 Department of Biochemistry & Molecular Biology, Howard University College of Medicine, Washington, WA, United States; 2 Department of Oncology, Georgetown University Medical Center, Washington, WA, United States; 3 Department of Biochemistry & Molecular Biology, Michigan State University, East Lansing, MI, United States

**Keywords:** epigenetics, molecular pathways, cancer, DNA regulation, RNA regulation

The field of cancer research has expanded beyond a sole focus on genetic mutations, increasingly acknowledging the significant role of epigenetics in the initiation, progression, and therapeutic resistance of cancer. Epigenetic mechanisms—heritable changes in gene expression that do not involve changes to the DNA sequence—are a vital layer of cellular regulation, and their disruption is a defining characteristic of cancer. This Research Topic, featuring seven contributing articles, was created to serve as a platform for diverse, advanced research and to summarize recent progress in understanding how epigenetic modifications drive the cancer development process. Our main goal was to discover new epigenetic markers and pathways that could be useful for cancer diagnosis, prognosis, and treatment.

This Research Topic was anchored by a comprehensive review by Sherif et al., which provides an overview of the key mechanisms and technologies currently explored in cancer epigenetics. This article also discusses other contributions by highlighting the established principles of DNA methylation, histone modification, and non-coding RNA regulation. Zhu et al. provided a focused review of the “transcriptomic era of cancers in females,” offering new epigenetic perspectives and therapeutic prospects, specifically for female-related malignancies. This study underscores the importance of sex-specific approaches to epigenetic research.

Several contributions in this Research Topic have examined specific epigenetic regulators and their roles as diagnostic or prognostic markers. The article by Zhao et al. provides a detailed analysis of the SWI/SNF complex, a key chromatin remodeler, and its high-frequency mutant subunits in various tumors. This study highlights how the fundamental epigenetic machinery is often dysregulated in cancer. Similarly, Lei et al. presented a meta-analysis of the prognostic significance of lncRNA FGD5-AS1 across different malignancies, suggesting its potential as a broad prognostic indicator. Additionally, Yin et al. identified EFTUD2 as a promising diagnostic and prognostic marker in lung adenocarcinoma, linking its involvement in the tumor immune microenvironment and glycolysis. Together, these studies demonstrate how a focused understanding of individual epigenetic factors can yield powerful insights into tumor progression.

The Research Topic also broadens its scope to include the influence of external factors and systemic consequences of malignancy. Yan et al. explored the relationship between the Hepatitis B virus X protein and the TGF-β pathway in the carcinogenesis of hepatocellular carcinoma, offering a unique perspective on how viral factors can engage with cellular signaling to drive cancer. Finally, Song et al. provided a scoping review of the genes related to cancer-related fatigue, a significant quality-of-life issue for patients. This contribution, while addressing a different facet of cancer, implicitly underscores the broad systemic impact of the disease, which is often mediated by epigenetic changes that regulate gene expression and cellular function.

**Figure 1:**
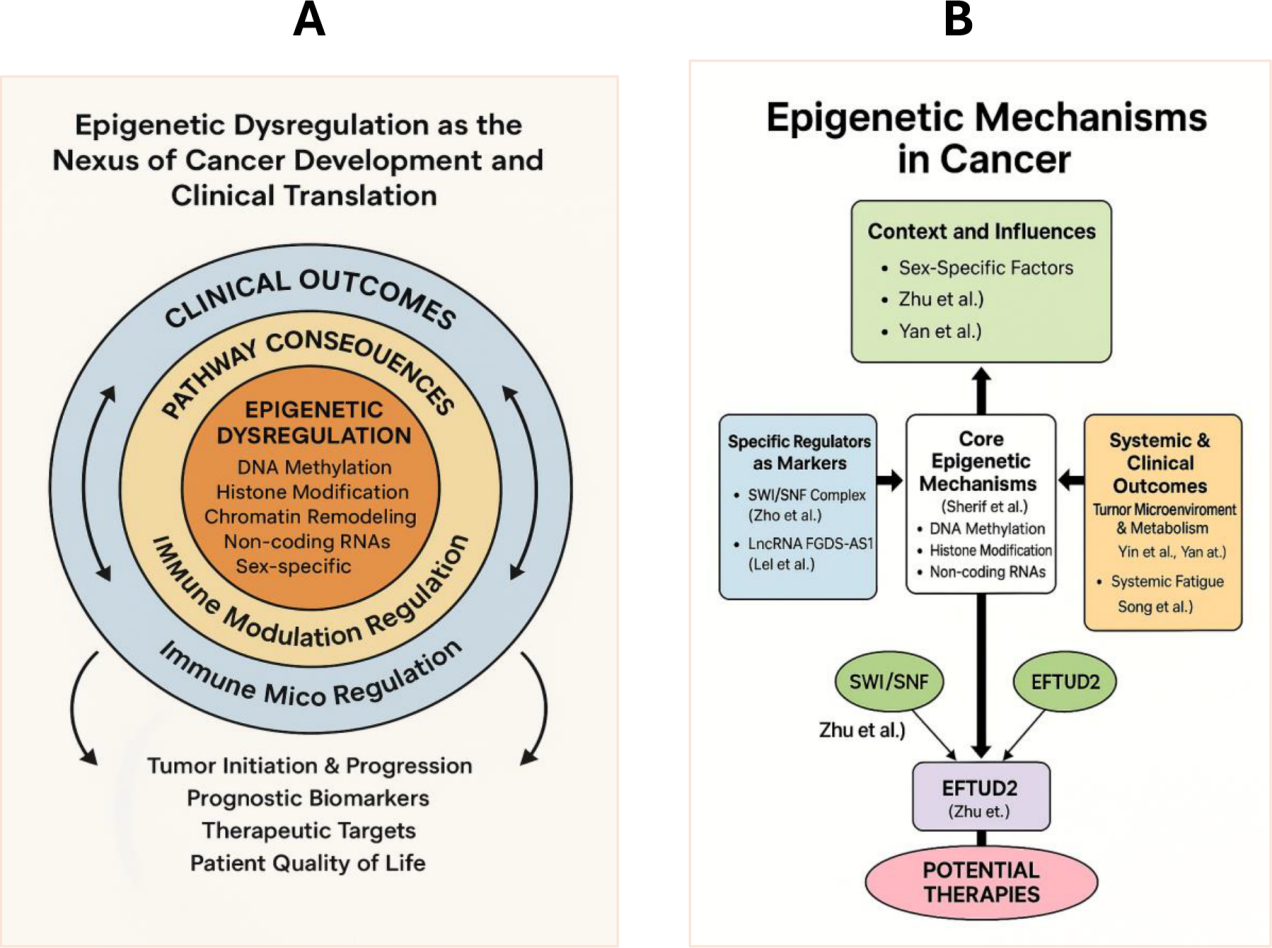
The Landscape of Epigenetic Regulation in Cancer and the Scope of the Research Topic. **(A)** Schematic representation of the nexus between epigenetic dysregulation and clinical translation. The core identifies fundamental molecular aberrations (DNA methylation, histone modification, chromatin remodeling, non-coding RNAs, and sex-specific factors) that drive systemic pathway consequences, specifically through immune modulation and micro-regulation. These processes culminate in real-world clinical outcomes, including tumor progression, the identification of prognostic biomarkers, and the development of potential therapeutic targets to improve patient quality of life. **(B)** Graphical summary of the contributions to this Research Topic. This collection comprises several original publications focusing on diverse “Epigenetic mechanisms in cancer,” ranging from mechanistic studies on chromatin remodeling to clinical investigations of epigenetic biomarkers. Together, these works highlight the interdisciplinary effort required to translate basic epigenetic discoveries into improved oncology care.

Taken together, the diverse and innovative findings presented in this article Research Topic place the field’s discoveries in a broader context. These articles move beyond descriptive observations to mechanistic investigations, bridging the gap between epigenetic dysregulation and functional outcomes in cancer. The insights gained from this Research Topic not only advance our fundamental understanding of cancer biology but also pave the way for the development of new diagnostic and prognostic markers, as well as novel epigenetic-based therapies (**Figure 1A**).

Future research should focus on translating these findings into clinical practice and exploring the crosstalk between epigenetic mechanisms as conceptualized in **Figure 1B**.

The Editors would like to extend their sincere gratitude to all the authors and reviewers for their outstanding contributions and dedication to this Research Topic. We are confident that this Research Topic will serve as a valuable resource and catalyst for further exploration in the dynamic and critical fields of cancer epigenetics.

